# Understanding the Mechanistic Contribution of Herbal Extracts in Compound Kushen Injection With Transcriptome Analysis

**DOI:** 10.3389/fonc.2019.00632

**Published:** 2019-07-12

**Authors:** Hanyuan Shen, Zhipeng Qu, Yuka Harata-Lee, Thazin Nwe Aung, Jian Cui, Wei Wang, R. Daniel Kortschak, David L. Adelson

**Affiliations:** ^1^Zhendong Australia-China Centre for Molecular Chinese Medicine, School of Biological Sciences, University of Adelaide, Adelaide, SA, Australia; ^2^Zhendong Research Institute, Shanxi-Zhendong Pharmaceutical Co., Ltd, Beijing, China

**Keywords:** transcriptome, herb combination, traditional Chinese medicine, cancer treatment, Compound Kushen Injection

## Abstract

Herbal compatibility is the knowledge of which herbs to combine in traditional Chinese medicine (TCM) formulations. The lack of understanding of herbal compatibility is one of the key problems for the application and popularization of TCM in western society. Because of the chemical complexity of herbal medicines, it is simpler to begin to conduct compatibility research based on herbs rather than component plant secondary metabolites. We have used transcriptome analysis to explore the effects and interactions of two plant extracts (Kushen and Baituling) combined in Compound Kushen Injection (CKI). Based on shared chemical compounds and *in vitro* cytotoxicity comparisons, we found that both the major compounds in CKI, and the cytotoxicity effects of CKI were mainly derived from the extract of Kushen (*Sophorae flavescentis*). We generated and analyzed transcriptome data from MDA-MB-231 cells treated with single-herb extracts or CKI and results showed that Kushen contributed to the perturbation of the majority of cytotoxicity/cancer related pathways in CKI such as cell cycle and DNA replication. We also found that Baituling (*Heterosmilax yunnanensis Gagnep*) could not only enhance the cytotoxic effects of Kushen in CKI, but also activate immune-related pathways. Our analyses predicted that IL-1β gene expression was upregulated by Baituling in CKI and we confirmed that IL-1β protein expression was increased using an ELISA assay. Altogether, these findings help to explain the rationale for combining Kushen and Baituling in CKI, and show that transcriptome analysis using single herb extracts is an effective method for understanding herbal compatibility in TCM.

## Introduction

At present many complex and chronic diseases rely on therapies that combine modern pharmaceuticals. A similar multiple-herb strategy known as “Fufang” is an essential component in traditional Chinese medicine (TCM) theory and is used to achieve better therapeutic results, and reduce side effects and herbal toxicity (1, 2). As a result of thousands of years' of accumulated clinical practice, TCM has more than 100,000 formulae and abundant experience that has contributed to an understanding of which herbs should be combined in particular circumstances, herein referred to as herbal compatibility (3, 4). However, because herbal medicines are made up of complex mixtures of plant secondary metabolites, the mechanism of most TCM formulas has not been explored. This limitation of TCM has become one of the key problems for its modernization, and hinders the application and popularization of herbal medicines (5).

Recent rapid developments in analytical chemistry and molecular biology have provided methods for researchers to tackle the complex mechanisms of herbal compatibility on a number of different levels. Usually, these methods focus on one or a few components within a complex mixture, in attempts to reveal how preparation/extraction for combined use can change their concentrations in products or pharmacokinetic processes *in vivo* (6–9). However, as a complex mixture may contain thousands of compounds, it is unclear how changing one or several components in a TCM formula can explain and account for the principles and observations of herbal compatibility. Furthermore, pharmacological models that measure phenotypes associated with efficacy or proxies for efficacy are limited in their ability to explain potential therapeutic effects and mechanisms. New high-throughput technologies for measuring molecular phenotypes such as gene expression, and bioinformatic methods can provide systematic ways for refining and clarifying complex biological processes that result from hundreds or thousands of molecular interactions. By applying these methods to the study of TCM, it is possible to transform the research paradigm from “main active compound that influences one target” to “multiple components that influence many network targets” (10, 11). Although it is now common to apply RNA-sequencing and systematic methods to study the effects of whole TCM formulae, no literature has applied these methods to study herbal compatibility. In this report, we apply transcriptome analysis to identify how the combination of Kushen (*Sophorae flavescentis*) and Baituling (*Heterosmilax yunnanensis Gagnep*) extracts can account for the broader and increased effects observed in Compound Kushen Injection (CKI).

Our model system for dissecting herbal compatibility, CKI, is derived from an ancient Chinese formula and was approved by the State Food and Drug Administration (SFDA) of China in 1995. It is widely used as an adjuvant medicine in the treatment of carcinomas for pain relief, activation of innate immune response and reduced side effects in cancer therapy (12, 13). Our previous results have shown that CKI suppresses the growth of cancer cells by inhibiting cell cycle, energy metabolism, and DNA repair pathways (10, 14). Kushen is considered to be the principal herb and major contributor to the molecular effects of CKI. Many published studies have reported on the alkaloids and flavonoids contained in CKI, most of which are extracted from Kushen. These compounds have been reported to have a variety of bioactivities, including antitumor, antioxidant, and anti-inflammatory activities (15, 16). However, there is no literature that mentions the role of Baituling in CKI. Therefore, current studies are not sufficient to provide a rational framework for CKI prescription or explain its molecular mechanisms.

In this report, we break down the formula into its individual components in order to study the herbal compatibility of CKI. By comparing transcriptome changes in MDA-MB-231 cells between CKI and single herbal extract treatments, we found that Kushen extract alone, perturbed most of the pathways through which CKI exerts its effects on cancer cells. However, integrating Kushen with Baituling can enhance the effects of Kushen alone on cancer-related pathways and in addition can activate innate immune functions. These results support the TCM rationale behind the CKI formula and confirm RNA-sequencing as a useful tool for the identification of candidate mechanisms in TCM research.

## Materials and Methods

### Cell Culture and Drugs

MDA-MB-231 (ATCC Cat# CRM-HTB-26, RRID:CVCL_0062), HepG2 (ATCC Cat# HB-8065, RRID:CVCL_0027) and A431 (ATCC Cat# CRL-7907, RRID:CVCL_0037) cells were purchased from ATCC (VA, USA). All cell lines were cultured at 37°C with 5% CO_2_ in DMEM (Thermo Fisher Scientific, MA, USA) with 10% fetal bovine serum (Thermo Fisher Scientific). CKI, Baituling, and Kushen injections were provided by Zhendong pharmaceutical Co.Ltd (China). Baituling and Kushen injections were manufactured using the same processes as CKI and diluted to the equivalent concentration of CKI (total alkaloid concentration at 2 mg/ml). The concentration at 2 mg/ml (total alkaloid concentration for CKI, and equivalent total alkaloid concentration for Kushen and Baituling injections) was used in all cell assays and transcriptome analysis.

All *in vitro* assays were conducted in 6-well or 96-well plates. The seeding density was 4 × 10^5^ for 6-well plates across all three cell lines. For 96-well plates, MDA-MB-231 cells, A431 cells and HepG2 cells were seeded at 1.6 × 10^5^cells/well, 8 × 10^4^ cells/well, and 4 × 10^3^cells/well respectively. Cells were cultured overnight before drug treatment and the treatment time was 48 h for all assays.

### Components Comparison With HPLC

CKI, Kushen or Baituling injection was diluted 1:20 with MilliQ water and then analyzed on a photodiode-array UV-Vis detector equipped Shimadzu HPLC (Japan) with a preparative C18 column (5 μm, 250 × 10 mm, Phenomenex, CA, USA). The recording range is from 200 to 280 nm, with monitoring at 215 nm. 0.01M ammonium acetate (adjusted to pH 8.0, solvent A) and acetonitrile + 0.09 % trifluoroacetic acid (solvent B) were used as mobile phase and flow rate is 2 ml/min with linear gradient elution (0 min, 100 % A; 60 min, 65 % A, 70 min, 100 % A). Nine Standard compounds, including Oxymatrine, Oxysophocarpine, N-methylcytisine, Matrine, Sophocarpine, Trifolirhizin, Adenine, Sophoridine (Beina Biotechnology Institute Co., Ltd, China), and macrozamin (Zhendong Pharmaceutical Co.Ltd, China), were used to characterize peaks in the HPLC profile.

### Cell Viability Assay

Cells were cultured and treated in 96-well plates. After 48 h drug treatment, 50 μl of XTT:PMS (at 1 mg/ml and 1.25 mM, respectively, and combined at 50:1 ratio, Sigma-Aldrich, MO, USA) was added into each well and incubated 4 h for the measurement of cell viability. A Biotrack II microplate reader was used to detect the absorbance at 492 nm.

### Apoptosis Rate With Cell Cycle Assay

After treatment, cells were harvested from 6-well plates and stained with propidium iodide (PI; Sigma-Aldrich) as previously described (17). The stained cells were quantified on a BD LSR Fortessa-X20 (BD Biosciences, NJ, USA) and the data were analyzed with FlowJo software (TreeStar Inc., OR, USA).

### qPCR for Transcriptome Validation

The assay was performed as previously described (10). The sequences of all primers are shown in the Supplementary Table.

### RNA Extraction and Sequencing

After treatment with injections, MDA-MB-231 cells were harvested from 6-well plates and snap-frozen with liquid nitrogen. Total RNA was isolated using the PureLink RNA mini kit (Thermo Fisher Scientific). Quality and quantity of RNAs were measured with a Bioanalyzer at the Cancer Genome Facility of the Australian Cancer Research Foundation (Australia) to ensure RIN>7.0 and sent to Novogene (China) for sequencing with paired-end 150 bp reads on an Illumina HiSeq X platform.

### Transcriptome Data Analysis

The adaptors and low-quality sequences in raw reads were trimmed with Trim_galore (v0.3.7, Babraham Bioinformatics) using parameters: —stringency 5 —paired. STAR (v2.5.3a) was used to align reads to reference genome (hg19, UCSC) with parameters: —outSAMstrandField intronMotif —outSAMattributes All —outFilterMismatchNmax 10 —seedSearchStartLmax 30(18). Differentially expressed genes were calculated with edgeR (v3.22.3) and selected with false discovery rate (FDR) < 0.05 and Log fold change >1 or < -1(19).

The GO and KEGG over-representation analyses were performed with ClueGO and visualized with Cytoscape v3.6.0 with following parameters: right-sided hypergeometric test for enrichment analysis; *p*-values were corrected for multiple testing according to the Benjamini-Hochberg method and biological process at 3rd level for GO terms (20, 21). The Signaling Pathway Impact Analysis (SPIA) package in R was used to conduct the pathway perturbation analysis using all DE genes (FDR < 0.05) (22). R Pathview package was used to visualize specific KEGG pathways (23). String (V11.0) was used to identify protein-protein interactions with a threshold of 0.4 for minimum interaction score (24).

### ELISA for IL-1β Level

A431 cells were treated with different injections for 48 h in 96-well plates and the cell culture supernatant was collected and tested for the level of IL-1**β** by ELISA using human interleukin-1 beta ELISA kit (Biosensis, CA, USA) according to the kit protocol. The absorbance at 450 nm was detected with Multiskan Ascent Plate Reader.

## Results

### HPLC Comparison of the Composition of CKI and Single Extract Injections

**Figure 1:**
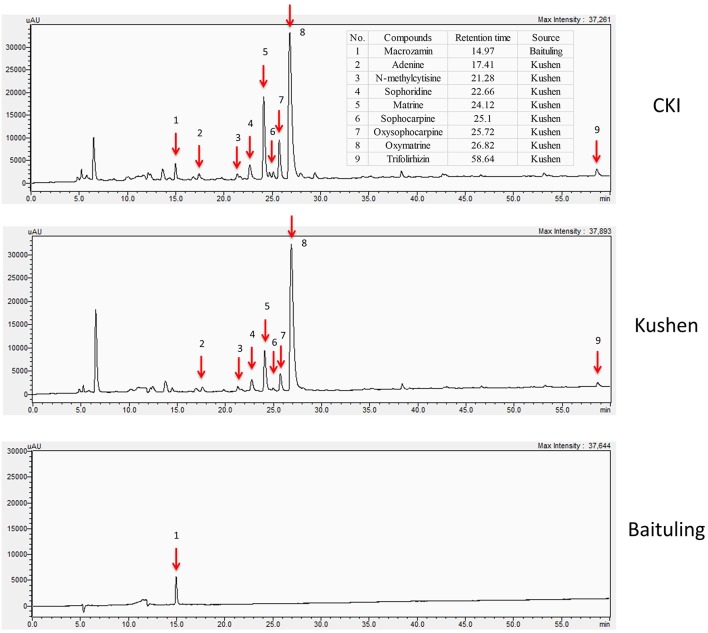
HPLC profiles of CKI, Kushen and Baituling injections. Nine component compounds characterized using standard compounds are marked with red arrows.

In order to obtain information about plant specific components in CKI, we used high-performance liquid chromatography (HPLC) to compare the compounds in CKI and two single herb extracts/injections (Figure 1). From the chromatographic profile, it can be seen that Kushen injection contributes most of the major chemical components in CKI. In contrast, few compounds were detected in Baituling injection, which only contributes one major compound to CKI. Based on comparison with 9 reference standard compounds, 8 main compounds derived from Kushen (adenine, N-methylcytisine, sophorodine, matrine, sophocarpine, oxysophocarpine, oxymatrine, and trifolirhizin) are shown to contribute to CKI. Macrozamin, which is used as a control marker for Baituling during manufacturing, only appeared in CKI and Baituling injection. These results indicate that CKI contains major compounds from both Kushen and Baituling, and Kushen contributes most of the major chemical components in CKI.

### Comparison of the Anticancer Effects Between CKI and Single Injections

**Figure 2:**
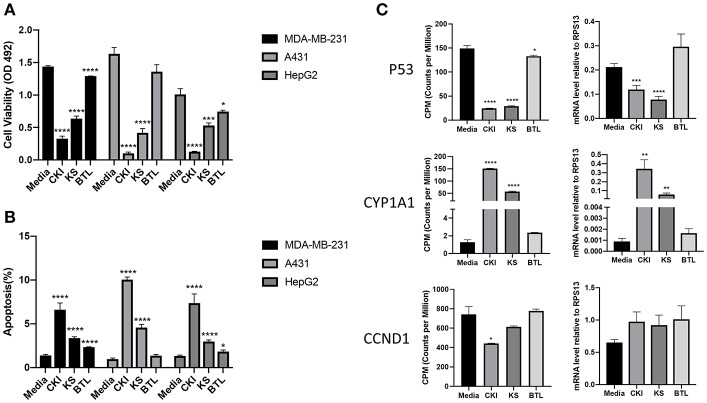
Comparison of the effect of CKI and single herb injections on cancer cell lines. **(A,B)** Viability and the percentage of apoptotic cells treated with different injections for 48 h. **(C)** q-PCR validation of RNA sequencing results. The expression value in transcriptome was normalized as CPM (counts per million) using a TMM (Trimmed-Mean of M values) methods from edgeR package. Results are represented as means ±SEM (*n* = 9). Statistical analyses were performed using the *t*-test compared to untreated “Media” (^*^p < 0.05, ^**^p < 0.01, ^***^p < 0.001, ^****^p < 0.0001).

Our previous results showed CKI suppressed the proliferation of and induced apoptosis in MCF-7 cells (10). To determine whether single injections had similar phenotypic effects as CKI, we conducted XTT assays to measure cell viability using three different cell lines; MDA-MB-231, A431 and HepG2. Results showed that Kushen had stronger cytotoxic effects than Baituling in all three cell lines. However, neither of the single injections had apoptotic effects comparable to CKI (Figure 2A) based on rates of apoptosis determined by flow cytometry with propidium iodide (PI) staining. Consistent with XTT cell viability results, more apoptotic cells were found in CKI than single injections treatments and Baituling had the smallest effect on apoptosis of the three injections (Figure 2B).

### Comparison of MDA-MB-231 Transcriptomes From CKI and Single Injection Treatments

In order to elucidate the molecular mechanisms of herbal compatibility in CKI, we carried out transcriptome profiling from CKI and the single injection treated MDA-MB-231 cells. Triplicate samples for each treatment clustered well in multidimensional scaling plots and different treatments were clearly separated (Supplementary Figure 1). Although CKI and Kushen injection have similar chemical profiles, the inclusion of Baituling in CKI is sufficient to change the transcriptome of MDA-MB-231 cells compared to Kushen single injection treatment. We used edgeR (19) to identify differentially expressed (DE) genes for each injection treatment compared to untreated. Only 253 DE genes were identified in Baituling treated cells, which is much less than CKI and Kushen treatments (Supplementary Figure 2). In addition, we also identified DE genes for CKI treatment compared to Kushen treatment to identify the effects of Baituling in CKI (Supplementary Table 3).

To validate the results of transcriptome analysis, we performed quantitative PCR for several genes known to be important read-outs for the effects of CKI; TP53, CYD1A1, and CCND1. Their expression levels confirmed the interaction of Baituling with Kushen observed in the overall RNA sequencing results (Figure 2C).

### Similar Effects of CKI and Kushen Single Injection on MDA-MB-231 Cells

**Figure 3:**
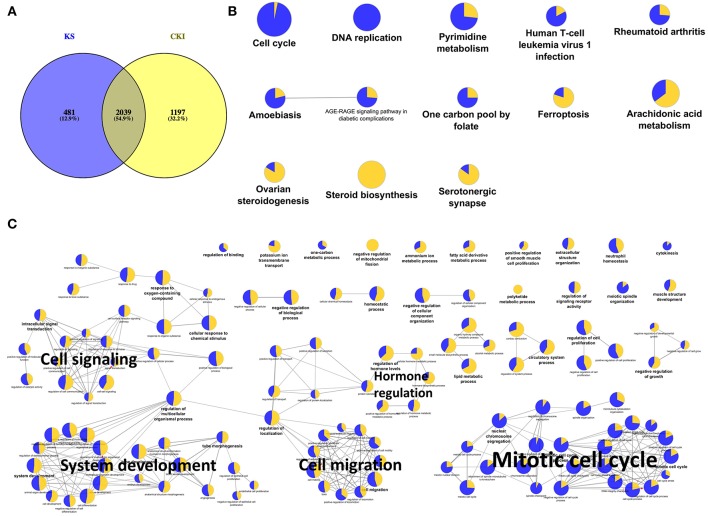
Significantly differentially expressed genes shared by Kushen (2 mg/ml) and CKI (2 mg/ml) treated cells and their functional enrichment analysis. **(A)** Venn diagram showing the number of differentially regulated genes in MDA-MB-231 cells treated with Kushen (KS; blue) or CKI (yellow) compared to untreated cells. **(B,C)** Over-represented KEGG pathways and GO terms (Biological Process at 3rd level, GO terms with FDR < 0.01 were showed and FDR < 0.05 were listed in Supplementary Table 5) for genes similarly regulated by Kushen and CKI. Node size is proportional to the statistical significance of over-representation and colors represent the proportion of up or down-regulated genes (yellow=up-regulated and blue=down-regulated). Similar GO terms are clustered (with representative terms shown in bold) and connected with edges.

Because Kushen is considered to be the primary active herb in CKI, and also we observed much more shared DE genes between CKI and Kushen treatments than between CKI and Baituling treatments (Supplementary Figure 2), we first examined the overlap of DE genes between Kushen and CKI. We identified 2,520 and 3,236 DE genes in cells treated with Kushen or CKI compared to untreated cells, respectively. By comparing these two DE gene sets, we identified 2,039 genes shared between the two groups (Figure 3A), indicating that Kushen contributed to the majority of effects from CKI.

In order to better understand the functions of shared DE genes between Kushen and CKI, we performed over-representation analysis using Gene Ontology (GO) and Kyoto Encyclopedia of Genes and Genomes (KEGG) pathways for these 2,039 genes (Figures 3B,C; Supplementary Table 5). The results showed that cell cycle- and DNA replication-related pathways and terms were largely down-regulated by both Kushen and CKI. Based on our previous publications, perturbed regulation of genes in these pathways and annotated by these terms was associated with the observed cell viability and apoptotic effects CKI on cancer cells (10, 14). Other terms showing perturbation of metabolic processes and cell migration identified in this study, such as “pyrimidine metabolism,” “steroid biosynthesis,” and “positive regulation of locomotion,” also showed up in our previous research on the effects of CKI (25). Altogether, these results indicated that Kushen was very important to the major molecular consequences of CKI treatment, and perturbed most of the biological functions perturbed by CKI, resulting in reduced viability and and increased apoptosis in cancer cells.

### Different Effects of CKI and Single Injections on MDA-MB-231 Cells

**Figure 4:**
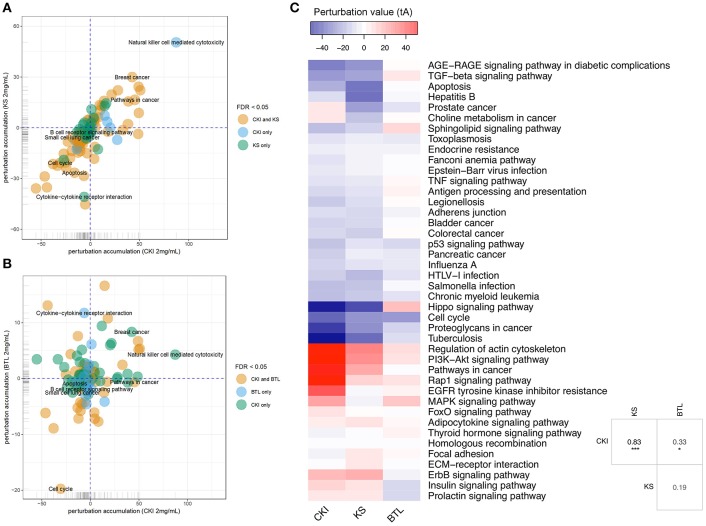
Pathway perturbation analysis for CKI and single injections on MDA-MB-231 cells. **(A,B)** Perturbation accumulation and significance of perturbation for different KEGG pathways treated with different injections. Positive perturbation accumulation values mean the pathway is activated and vice versa. Dot colors indicate whether the pathway was significantly perturbed by CKI and/or Kushen. **(C)** Heatmap showing the perturbation value of shared significantly perturbed pathways for the three injections. Table on the right-bottom corner shows the correlation coefficients for the perturbation value for the three injections. “^*^” and “^***^” represent *p* < 0.05 and *p* < 0.001 respectively (Pearson's correlation test).

Although the above results for enrichment analysis showed that Kushen and CKI mainly regulate the same pathways, they did not specifically show the magnitude and direction of these perturbations. We, therefore, performed Signaling Pathway Impact Analysis (SPIA) to compare the significantly perturbed functional pathways across the different treatments. Ninety-two pathways were found to be significantly perturbed by CKI while only 30 of them were shown to be activated. For Kushen and Baituling, the ratios of activated/inhibited were 29/100 and 24/58, respectively (Supplementary Table 4). Clearly, Baituling perturbed fewer pathways, but the ratio of activated/inhibited was higher than for Kushen or CKI. Interestingly, most pathways were perturbed in the same way (inhibited or activated) by CKI and Kushen as shown in Figures 4A,B with very few pathways showing different directions of perturbation.

**Figure 5:**
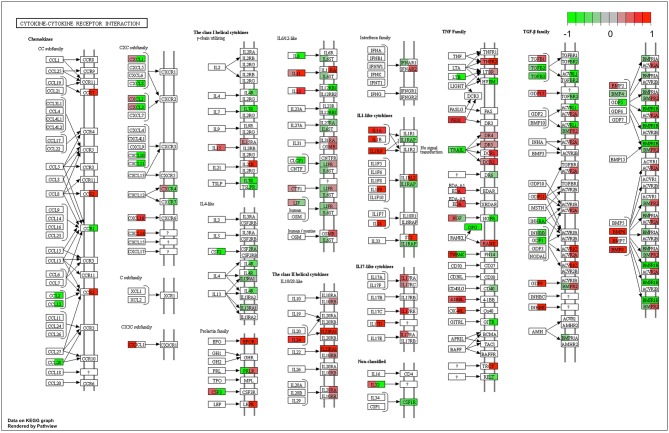
Comparison of gene expression changes caused by Baituling and Kushen treatments in the cytokine-cytokine receptor interaction pathway. Left half of each box represents the gene expression change with Baituling treatment and the right half represents the effect of Kushen treatment. White or gray colors indicate no significant change in gene expression as a function of treatment.

This similarity of effects between Kushen and CKI could also be seen in the high level of correlation for pathway perturbation between Kushen and CKI (0.83 correlation coefficient) compared to (0.33 correlation coefficient) for Baituling and CKI. CKI also had stronger perturbation effects on most pathways (Figure 4C). In pathways contributing to cytotoxic effects in cancer cells, such as cell cycle, p53 signaling pathway, proteoglycans in cancer and pathways in cancer, Baituling perturbed the pathways in the same direction as Kushen and seemed to reinforce those effects in CKI. However, the cytokine-cytokine receptor interaction pathway was very interesting as it was perturbed in an opposite fashion in Baituling compared to Kushen and did not show up as significantly perturbed by CKI treatment (Supplementary Figure 3). By comparing DE genes for Baituling and Kushen in the cytokine-cytokine receptor interaction pathway (Figure 5), we observed that many genes were oppositely regulated by the two single injections, such as genes in the CXC subfamily and IL6/12-like cytokine and receptor genes, which supported the SPIA results.

### The Functions of Baituling in CKI

**Figure 6:**
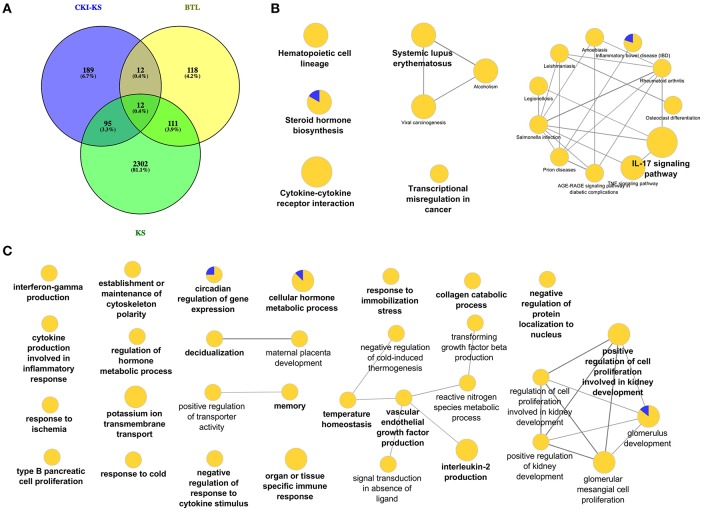
DE genes regulated by Baituling in MDA-MB-231 cells line and their functional enrichment analysis. **(A)** Venn diagram showing the number of differentially regulated genes with CKI compared to Kushen (CKI-KS—Blue) and single herb injections (KS—Green or BTL—Yellow) compared to untreated. **(B,C)** Over-represented KEGG pathways and GO terms (Biological Process at 3rd level) for DE genes calculated by CKI compared to Kushen treated. Node size is proportional to the statistical significance of over-representation and colors represent the proportion of up or down-regulated genes (yellow=up-regulated and blue=down-regulated). Terms are clustered based on similar GO group (shown in bold) and related ones are connected with edges.

In order to investigate the function of Baituling in CKI, we identified the DE genes of CKI treatment compare to Kushen treatment. Only 308 DE genes were found (Figure 6A). KEGG analysis of these genes showed that with the exception of steroid hormone biosynthesis and transcriptional misregulation in cancer, all other pathways were related to immune function, and most genes in this set were up-regulated (Figure 6B, Supplementary Table 5). This result was consistent with findings in the SPIA analysis that Baituling tended to activate immune-related pathways. The over-represented GO terms for this gene set also included interferon-gamma production, organ or tissue specific immune response and interleukin-2 production, all aspects of immune function and which only contained up-regulated DE genes (Figure 6C).

**Figure 7:**
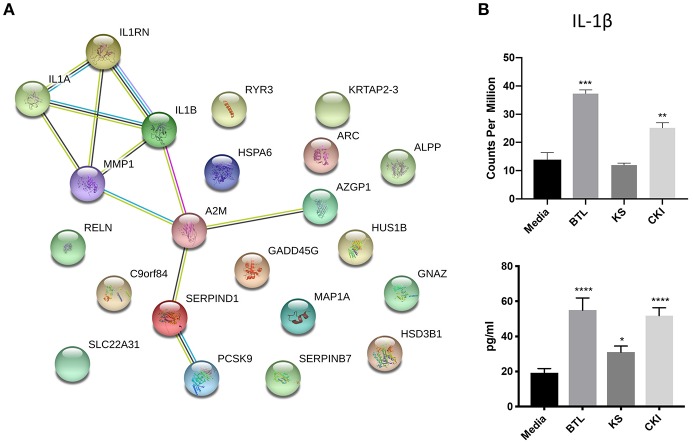
Validation of IL-1β expression changes regulated by Baituling. **(A)** Diagram showing protein-protein interactions for common DE genes between CKI compared to Kushen, and Baituling compared to untreated. **(B)** Comparison of expression levels and protein concentration for IL-1β with different treatments. Top panel; IL-1β gene expression levels determined by RNA sequencing, bottom panel; levels of IL-1β in culture supernatant measured by ELISA. Statistical analyses were performed using the *t*-test compared to untreated “Media” (^*^p < 0.05, ^**^p < 0.01, ^***^p < 0.001, ^****^p < 0.0001).

The genes in the common set between Kushen and CKI compared to Kushen (Figure 6A) were originally changed with Kushen treatment and then further significantly regulated in combination with Baituling (107 genes). Only three pathways (IL-17 signaling, salmonella infection, and steroid hormone biosynthesis) were over-expressed by genes in this set, indicating Baituling could also modify functions related to immune system and hormone function upregulated by Kushen in CKI (Supplementary Figure 4). Furthermore, 24 genes appeared in both Baituling and CKI compared to Kushen set (Figure 6A), which can be regarded as the direct contribution from Baituling to the effects of CKI. Analysis of known protein-protein interactions in this gene set identified the IL-1 family and interacting proteins known to modulate immune function (Figure 7A). To verify that IL-1 protein levels were also changing as predicted, we carried out an ELISA assay to measure the IL-1β beta levels in different treatments. We were able to demonstrate that the increased IL-1β level in CKI treatment compared to untreated was mainly related to the effects of Baituling (Figure 7B). Altogether, these results showed that Baituling contributed to the effects of CKI primarily by altering functions related to the immune system.

In summary, we characterized the herbal compatibility of Kushen and Baitulin in CKI by comparing the individual effects of the herbal extracts to the combined extract using transcriptome analysis. In this fashion, we were able to explain the origin of CKI's different effects in MDA-MB-231 cells. In addition, we also showed that Baituling could enhance the reduction of cell viability and increased apoptosis effects from Kushen in CKI. These results not only explained the specific molecular basis of the TCM rationale of combining Kushen with Baituling but also illustrate a general method to apply transcriptome analysis to study herbal compatibility in TCM.

## Discussion

It is undeniable that chemical composition is the basis of therapeutic effects from herbal TCM. However, because the identification and quantification of all compounds for even a single herb are still extremely difficult if not impossible, there is a pressing need for alternative methods to conduct TCM research, particularly with respect to the study of herbal compatibility (26, 27). Herbal compatibility has a basis in TCM theory, but TCM theory is not generally accepted in Western medicine and it is difficult to map concepts from TCM theory to Western medicine. Methods that can identify the molecular consequences of TCM formulations and individual herbs can begin to provide such a map. One view of TCM is that it perturbs multiple targets or pathways with multiple low activity components to generate relatively strong effects. This is in contrast to the standard approach for pharmaceutical drug development which seeks to identify single compounds that inhibit a single pathway or target. However, this method of using one or several active compounds as representative of single herbs is problematic (11) for understanding the roles of individual plant extracts in TCM formulations. This is illustrated by our results; although containing similar amounts of the main chemical compounds, CKI has much stronger effects than Kushen extract alone. Therefore, a formula disassembly approach that uses single herbs is a practical way to study herbal compatibility. Combined with omics techniques and network analysis, we can represent the mechanisms of TCM herbal compatibility as interactions between target networks familiar to Western medicine. In this report, we took CKI, a prescription containing only two herbs, as a proof of principle. However, related methods can easily be applied to more complex formulae.

As a frequently used herb, and because it is considered to contain the main bioactive components in CKI, Kushen or its main alkaloids and flavonoids are commonly used to represent CKI in studies (28, 29). Furthermore, TCM theory also regards Kushen as the primary herb in CKI. Our results support these hypotheses and TCM theory at different levels. First, at the chemical level, HPLC profiles showed that the source of most major components in CKI is Kushen. Second, in terms of overall efficacy, Kushen has much stronger cytotoxic effects than Baituling on various cell lines. Third, at the gene level, a high proportion of DE genes regulated by Kushen or CKI are the same, and most of them are consistently up- or down-regulated. Furthermore, important genes are also regulated both by Kushen and CKI. Cytochrome P450 family 1 subfamily A member 1 (CYP1A1) gene, a steroid metabolizing enzyme which is important for steroid hormone responsive cancers and shown as the most over-expressed gene with CKI in our previous results, is also highly overexpressed by Kushen but not Baituling (30, 31). Also, the down-regulation of the TP53 gene primarily results from Kushen. Our previous results based on CKI treated cells underwent apoptosis while expression of TP53 decreased, which indicates the apoptosis induced by Kushen and CKI is not TP53-dependent (10). Finally, GO and KEGG over-representation analysis also indicated that the genes in major cancer related pathways and terms perturbed by CKI, including cell cycle, DNA replication, and cell migration, are induced by the shared DE gene set between Kushen and CKI. Taken together, our findings agree with TCM theory which considers Kushen as the principle herb in CKI, and they map this specific part of TCM theory to effects on specific genetic networks and pathways.

On the other hand, we could find no Baituling literature and studies on macrozamin to support its application in CKI (32). From our HPLC and cytotoxic assay results, Baituling extract does not contain many components and has no significant effects on cancer cells. In addition, RNAseq analysis of Baituling-treated samples are close to the untreated samples in the multidimensional scaling plot, and only 253 DE genes compared to untreated were detected. However, after comparing the differences between CKI and Kushen, we found Baituling has a strong reinforcing effect on Kushen. The SPIA results showed Baituling can enhance many pathways' perturbation strength compared to Kushen treatment, including cell cycle, pathways in cancer and proteoglycans in cancer. In addition, many DE genes in immune-related pathways and GO terms are over-represented in CKI compared to Kushen. Together with the opposing direction of perturbation for the cytokine-cytokine receptor interaction pathway caused by Kushen and Baituling, we can conclude Baituling may also contribute to the immune regulatory effects of CKI. This was confirmed by our measurements of IL-1**β**, which was significantly up-regulated by Baituling and CKI. In summary, our results indicate that Baituling, an adjuvant herb in CKI according to TCM theory, may enhance the anticancer effects of Kushen and contribute to immune regulation.

In conclusion, we have explained the TCM herbal compatibility of CKI in the context of pathway perturbations using transcriptome analysis. Kushen primarily contributes to CKI effects on cancer cells by perturbing cell cycle regulation and other functions (10, 14), whereas Baituling enhanced potential anticancer effects for Kushen and activated the immune system. Therefore, the two herbs in CKI have complementary effects, in accordance with formulation theory in TCM. Compared to previous studies on herbal compatibility, our method can explain the beneficial interaction pattern of herbs in TCM formulae in a more systematic and comprehensive fashion.

## Data Availability

Data were submitted to NCBI Gene Expression Omnibus (GEO), with Accession Number GSE130358 and GSE130359, available at https://www.ncbi.nlm.nih.gov/geo/query/acc.cgi?acc=GSE130358 and https://www.ncbi.nlm.nih.gov/geo/query/acc.cgi?acc=GSE130359.

## Author Contributions

HS, ZQ, YH-L, and DA contributed to conception and design of the study. HS conducted the experiments and analyzed the data. TA and JC assisted the experiments. WW provided the injections used in the study. HS, RK, and DA wrote the manuscript. All authors contributed to manuscript revision, read and approved the submitted version.

### Conflict of Interest Statement

While a generous donation was used to set up the Zhendong Centre by Shanxi Zhendong Pharmaceutical Co., Ltd, they did not determine the research direction for this work or influence the analysis of the data. WW: is an employee of Zhendong Pharma seconded to Zhendong Centre to learn bioinformatics methods, DA: Director of the Zhendong Centre which was set up with a generous donation from the Zhendong Pharmaceutical Co., Ltd. Zhendong Pharmaceutical has had no control over these experiments, their design or analysis and have not exercised any editorial control over the manuscript. The remaining authors declare that the research was conducted in the absence of any commercial or financial relationships that could be construed as a potential conflict of interest.

## Supplementary Material

Supplementary Figure 1 Multiple dimensional scaling (MDS) plot for samples based on expression profiles of all genes (Untreated in black, Baituling in blue, Kushen in green, and CKI in blue).

Supplementary Figure 2 Venn diagram showing the number of differentially regulated genes in MDA-MB-231 cells treated with CKI (blue), Baituling (BTL, yellow), and Kushen (KS, green).

Supplementary Figure 3 Heatmap showing the perturbation value of significantly perturbed pathways only for one or two injections.

Supplementary Figure 4 Over-represented KEGG pathways and their contained genes showing shared DE genes between CKI (DE calculated by comparison to Kushen treated) and Kushen (DE calculated by comparison to untreated). Node size is proportional to the statistical significance of over-representation and genes are connected to their belonged pathways with edges.

Supplementary Table 1 Mapping rates for each RNA-seq result.

Supplementary Table 2 Primer sequences for qRT-PCR target genes.

Supplementary Table 3 DE gene lists for different comparisons.

Supplementary Table 4 SPIA results of significantly perturbed KEGG pathways for three injections.

Supplementary Table 5 Significantly over-represented GO and KEGG terms (count > 4 and *P*-value < 0.05).

- Sheet 1-2. GO and KEGG enrichment of DE genes shared by CKI compared to “untreated” and Kushen compared to “untreated.”
- Sheet 3-4. GO and KEGG enrichment of DE genes for CKI compared to Kushen.

## References

[1] Huang J Tang X Ye F He J Kong X. Clinical therapeutic effects of aspirin in combination with fufang danshen diwan, a traditional chinese medicine formula, on coronary heart disease: a systematic review and meta-analysis. Cell Physiol Biochem. (2016) 39:1955–63. 10.1159/000447892 27771711

[2] Hu X-Q Sun Y Lau E Zhao M Su S-B. Advances in synergistic combinations of chinese herbal medicine for the treatment of cancer. Curr Cancer Drug Targets. (2016) 16:346–56. 10.2174/1568009616666151207105851 26638885 PMC5425653

[3] Wang S Hu Y Tan W Wu X Chen R Cao J. Compatibility art of traditional Chinese medicine: from the perspective of herb pairs. J Ethnopharmacol. (2012) 143:412–23. 10.1016/j.jep.2012.07.033 22871585

[4] Zhou M Hong Y Lin X Shen L Feng Y. Recent pharmaceutical evidence on the compatibility rationality of traditional Chinese medicine. J Ethnopharmacol. (2017) 206:363–75. 10.1016/j.jep.2017.06.007 28606807

[5] Cai F-F Zhou W-J Wu R Su S-B. Systems biology approaches in the study of Chinese herbal formulae. Chin Med. (2018) 13:65. 10.1186/s13020-018-0221-x 30619503 PMC6311004

[6] Jin Y Tang Y-P Zhu Z-H Shang E-X Pang H-Q Shi X-Q. Pharmacokinetic comparison of seven major bio-active components in normal and blood stasis rats after oral administration of herb pair danggui-honghua by UPLC-TQ/MS. Molecules. (2017) 22:1746. 10.3390/molecules22101746 29039793 PMC6151798

[7] Liu P Shang E-X Zhu Y Qian D-W Duan J-A. Volatile component interaction effects on compatibility of Cyperi Rhizoma and Angelicae Sinensis Radix or Chuanxiong Rhizoma by UPLC-MS/MS and response surface analysis. J Pharm Biomed Anal. (2018) 160:135–43. 10.1016/j.jpba.2018.07.060 30086506

[8] Zhang Y Zhang Z Song R. The influence of compatibility of rhubarb and radix scutellariae on the pharmacokinetics of anthraquinones and flavonoids in rat plasma. Eur J Drug Metab Pharmacokinet. (2018) 43:291–300. 10.1007/s13318-017-0444-8 29134502

[9] Zhou J Cai H Tu S Duan Y Pei K Xu Y. Identification and analysis of compound profiles of sinisan based on “individual herb, herb-pair, herbal formula” before and after Processing Using UHPLC-Q-TOF/MS coupled with multiple statistical strategy. Molecules. (2018) 23:3128. 10.3390/molecules23123128 30501046 PMC6320893

[10] Qu Z Cui J Harata-Lee Y Aung TN Feng Q Raison JM. Identification of candidate anti-cancer molecular mechanisms of Compound Kushen Injection using functional genomics. Oncotarget. (2016) 7:66003–19. 10.18632/oncotarget.11788 27602759 PMC5323210

[11] Lv C Wu X Wang X Su J Zeng H Zhao J. The gene expression profiles in response to 102 traditional Chinese medicine (TCM) components: a general template for research on TCMs. Sci Rep. (2017) 7:352. 10.1038/s41598-017-00535-8 28336967 PMC5428649

[12] Ma X Li R-S Wang J Huang Y-Q Li P-Y Wang J. The therapeutic efficacy and safety of compound kushen injection combined with transarterial chemoembolization in unresectable hepatocellular carcinoma: an update systematic review and meta-analysis. Front Pharmacol. (2016) 7:70. 10.3389/fphar.2016.00070 27065861 PMC4814457

[13] Ao M Xiao X Li Q. Efficacy and safety of compound Kushen injection combined with chemotherapy on postoperative Patients with breast cancer: a meta-analysis of randomized controlled trials. Medicine. (2019) 98:e14024. 10.1097/MD.0000000000014024 30653109 PMC6370033

[14] Cui J Qu Z Harata-Lee Y Nwe Aung T Shen H Wang W. Cell cycle, energy metabolism and DNA repair pathways in cancer cells are suppressed by Compound Kushen Injection. BMC Cancer. (2019) 19:103. 10.1186/s12885-018-5230-8 30678652 PMC6345000

[15] Hu H Wang S Zhang C Wang L Ding L Zhang J. Synthesis and *in vitro* inhibitory activity of matrine derivatives towards pro-inflammatory cytokines. Bioorg Med Chem Lett. (2010) 20:7537–9. 10.1016/j.bmcl.2010.09.075 21036613

[16] Wang W You R-L Qin W-J Hai L-N Fang M-J Huang G-H. Anti-tumor activities of active ingredients in Compound Kushen Injection. Acta Pharmacol Sin. (2015) 36:676–9. 10.1038/aps.2015.24 25982630 PMC4594177

[17] Riccardi C Nicoletti I. Analysis of apoptosis by propidium iodide staining and flow cytometry. Nat Protoc. (2006) 1:1458–61. 10.1038/nprot.2006.238 17406435

[18] Dobin A Davis CA Schlesinger F Drenkow J Zaleski C Jha S. STAR: ultrafast universal RNA-seq aligner. Bioinformatics. (2013) 29:15–21. 10.1093/bioinformatics/bts635 23104886 PMC3530905

[19] Robinson MD McCarthy DJ Smyth GK. edgeR: a Bioconductor package for differential expression analysis of digital gene expression data. Bioinformatics. (2010) 26:139–40. 10.1093/bioinformatics/btp616 19910308 PMC2796818

[20] Shannon P Markiel A Ozier O Baliga NS Wang JT Ramage D. Cytoscape: a software environment for integrated models of biomolecular interaction networks. Genome Res. (2003) 13:2498–504. 10.1101/gr.1239303 14597658 PMC403769

[21] Bindea G Mlecnik B Hackl H Charoentong P Tosolini M Kirilovsky A. ClueGO: a Cytoscape plug-in to decipher functionally grouped gene ontology and pathway annotation networks. Bioinformatics. (2009) 25:1091–3. 10.1093/bioinformatics/btp101 19237447 PMC2666812

[22] Tarca AL Draghici S Khatri P Hassan SS Mittal P Kim J-S. A novel signaling pathway impact analysis. Bioinformatics. (2008) 25:75–82. 10.1093/bioinformatics/btn577 18990722 PMC2732297

[23] Luo W Brouwer C. Pathview: an R/Bioconductor package for pathway-based data integration and visualization. Bioinformatics. (2013) 29:1830–1. 10.1093/bioinformatics/btt285 23740750 PMC3702256

[24] Jensen LJ Kuhn M Stark M Chaffron S Creevey C Muller J. STRING 8–a global view on proteins and their functional interactions in 630 organisms. Nucleic Acids Res. (2009) 37:D412–D416. 10.1093/nar/gkn760 18940858 PMC2686466

[25] Nourmohammadi S Aung TN Cui J Pei JV De Ieso ML Harata-Lee Y. Effect of Compound Kushen Injection, a natural compound mixture, and its identified chemical components on migration and invasion of colon, brain and breast cancer cell lines. Front Oncol. (2019) 9:314. 10.3389/fonc.2019.00314 31106149 PMC6498862

[26] He X-R Li C-G Zhu X-S Li Y-Q Jarouche M Bensoussan A. High-performance liquid chromatography coupled with tandem mass spectrometry technology in the analysis of Chinese Medicine Formulas: a bibliometric analysis (1997-2015). J Sep Sci. (2017) 40:81–92. 10.1002/jssc.201600784 27731929

[27] Huang T Zhong LLD Lin C-Y Zhao L Ning Z-W Hu D-D. Approaches in studying the pharmacology of Chinese Medicine formulas: bottom-up, top-down-and meeting in the middle. Chin Med. (2018) 13:15. 10.1186/s13020-018-0170-4 29588653 PMC5863461

[28] Gao L Wang K-X Zhou Y-Z Fang J-S Qin X-M Du G-H. Uncovering the anticancer mechanism of Compound Kushen Injection against HCC by integrating quantitative analysis, network analysis and experimental validation. Sci Rep. (2018) 8:624. 10.1038/s41598-017-18325-7 29330507 PMC5766629

[29] Jin Y Yang Q Liang L Ding L Liang Y Zhang D. Compound kushen injection suppresses human acute myeloid leukaemia by regulating the Prdxs/ROS/Trx1 signalling pathway. J Exp Clin Cancer Res. (2018) 37:277. 10.1186/s13046-018-0948-3 30454068 PMC6245615

[30] Nguyen CH Brenner S Huttary N Atanasov AG Dirsch VM Chatuphonprasert W. AHR/CYP1A1 interplay triggers lymphatic barrier breaching in breast cancer spheroids by inducing 12(S)-HETE synthesis. Hum Mol Genet. (2016) 25:5006–16. 10.1093/hmg/ddw329 28171546

[31] Kim J Cho YA Lee J Oh JH Sohn DK Chang HJ Abstract 2232: Dietary flavonoids, CYP1A1 genetic variants, and the risk of colorectal cancer. Cancer Res. (2017) 77:2232–2232. 10.1158/1538-7445.AM2017-2232 PMC5427897 28273931

[32] Lythgoe B Riggs NV. Macrozamin, a toxic nitrogen-containing primeveroside. Experientia. (1949) 5:471. 10.1007/BF02165424 15397410

[33] Shen H Qu Z Harata-Lee Y Nwe Aung T Cui J Wang W Understanding the Mechanistic Contribution of Herbal Extracts in Compound Kushen Injection with Transcriptome Analysis. bioRxiv. (2019). 10.1101/592964 PMC6660286 31380274

