# Supplementary figures and images for: Understanding the Mechanistic Contribution of Herbal Extracts in Compound Kushen Injection With Transcriptome Analysis
Source: Front Oncol. 2019 Jul 12;9:632. doi: 10.3389/fonc.2019.00632 (PMC6660286; doi:10.3389/fonc.2019.00632)

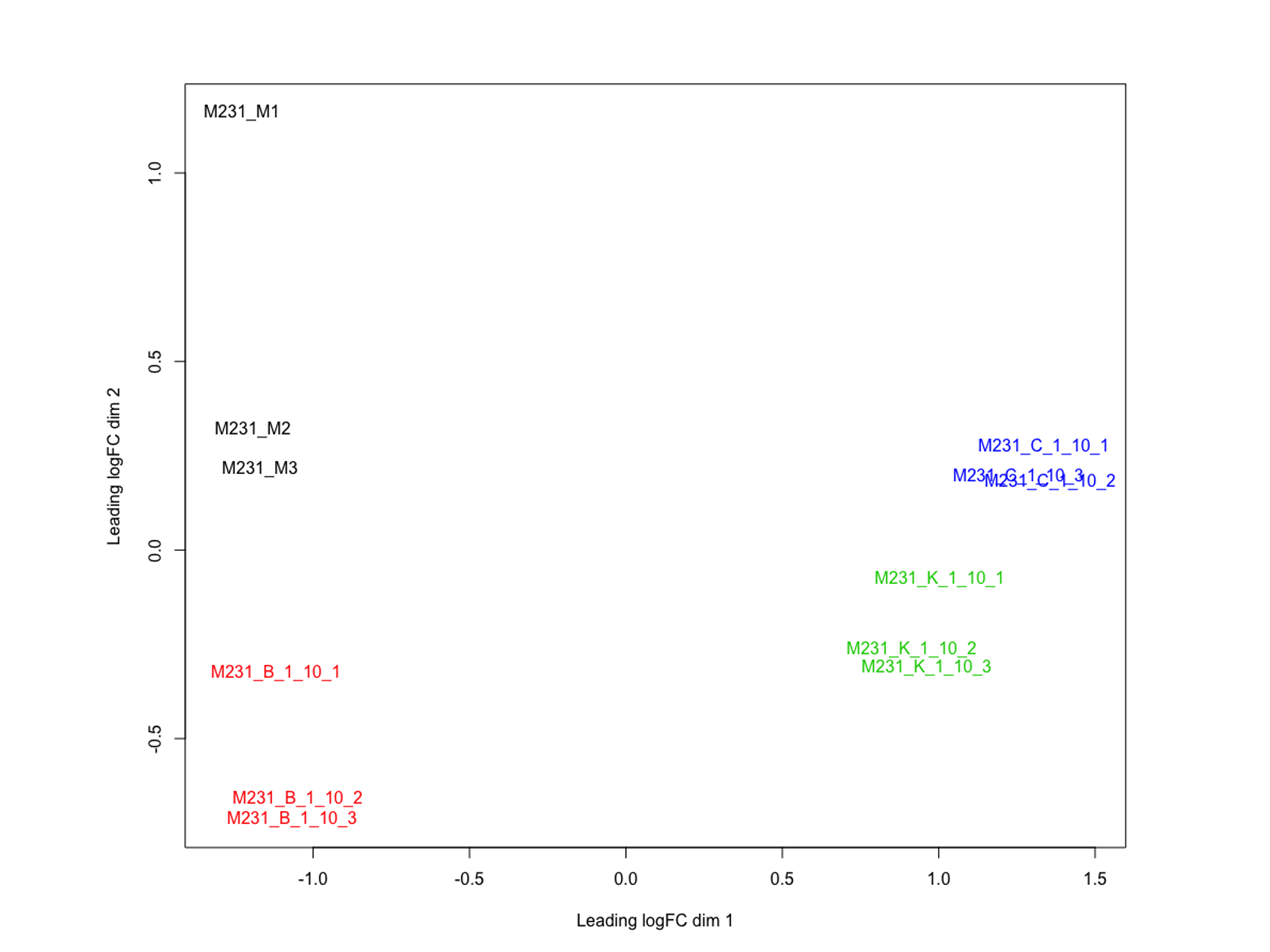

Supplement: Supplementary Figure 1 — Multiple dimensional scaling (MDS) plot for samples based on expression profiles of all genes (Untreated in black, Baituling in blue, Kushen in green, and CKI in blue). [file Image_1.TIF]

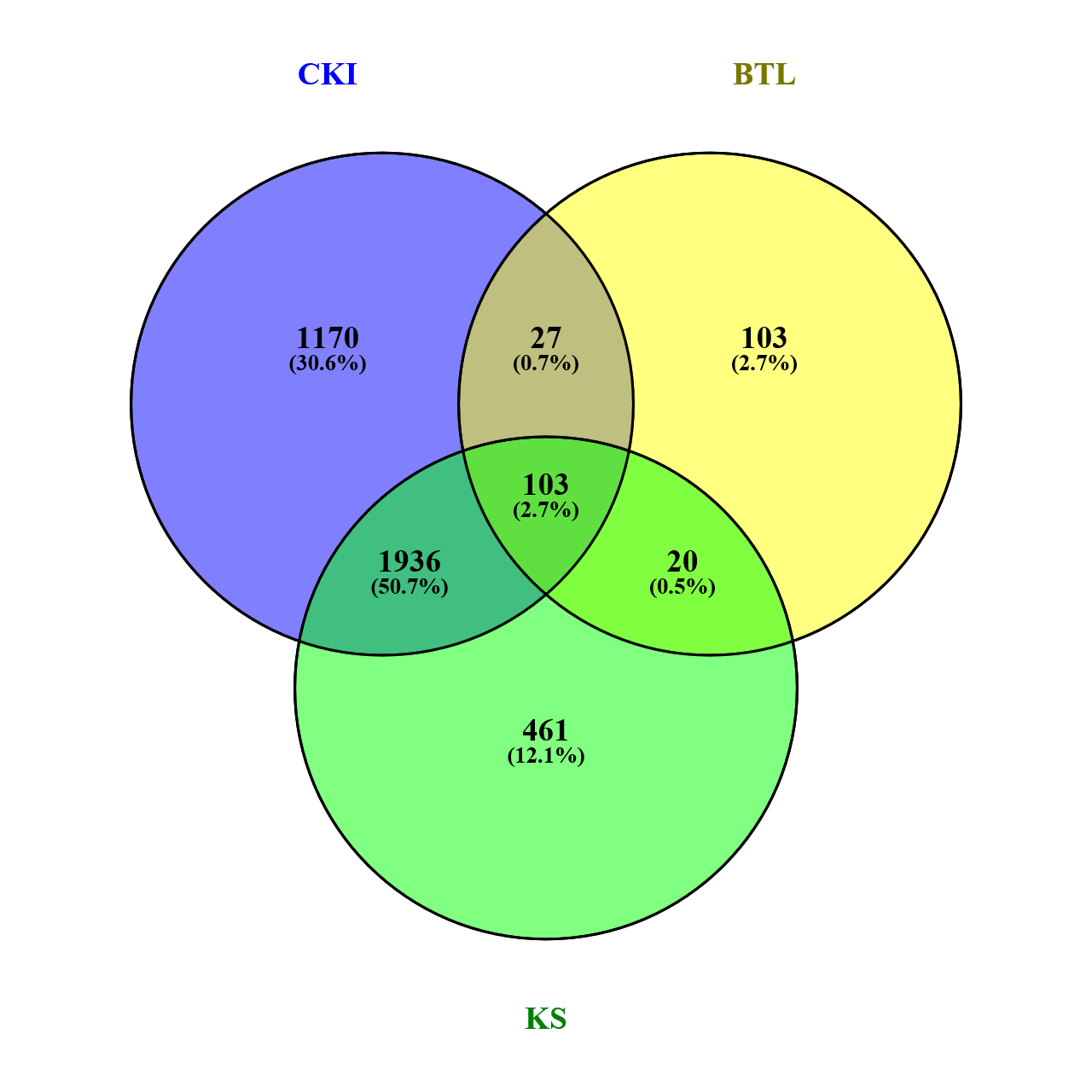

Supplement: Supplementary Figure 2 — Venn diagram showing the number of differentially regulated genes in MDA-MB-231 cells treated with CKI (blue), Baituling (BTL, yellow), and Kushen (KS, green). [file Image_2.PNG]

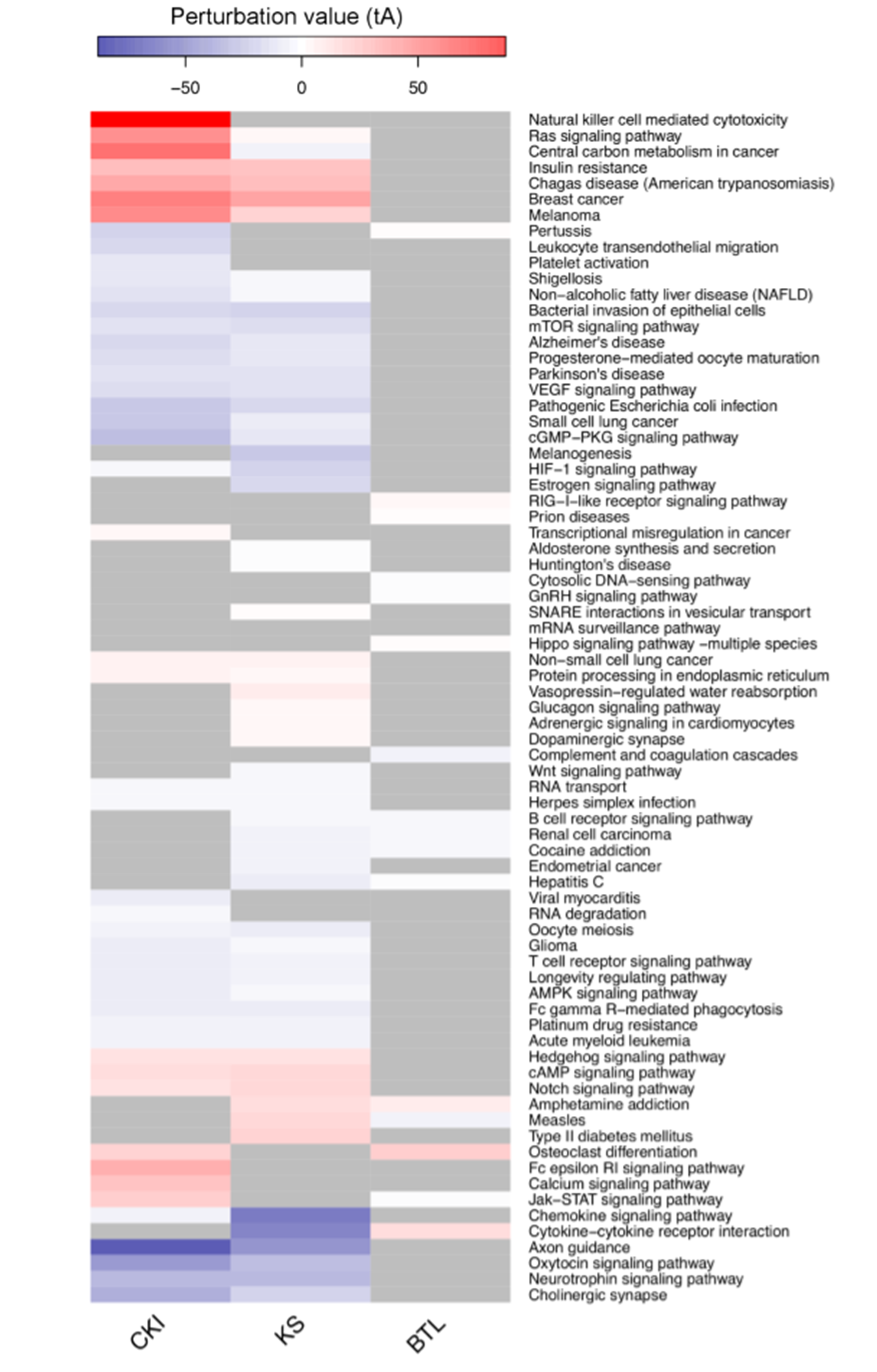

Supplement: Supplementary Figure 3 — Heatmap showing the perturbation value of significantly perturbed pathways only for one or two injections. [file Image_3.TIF]

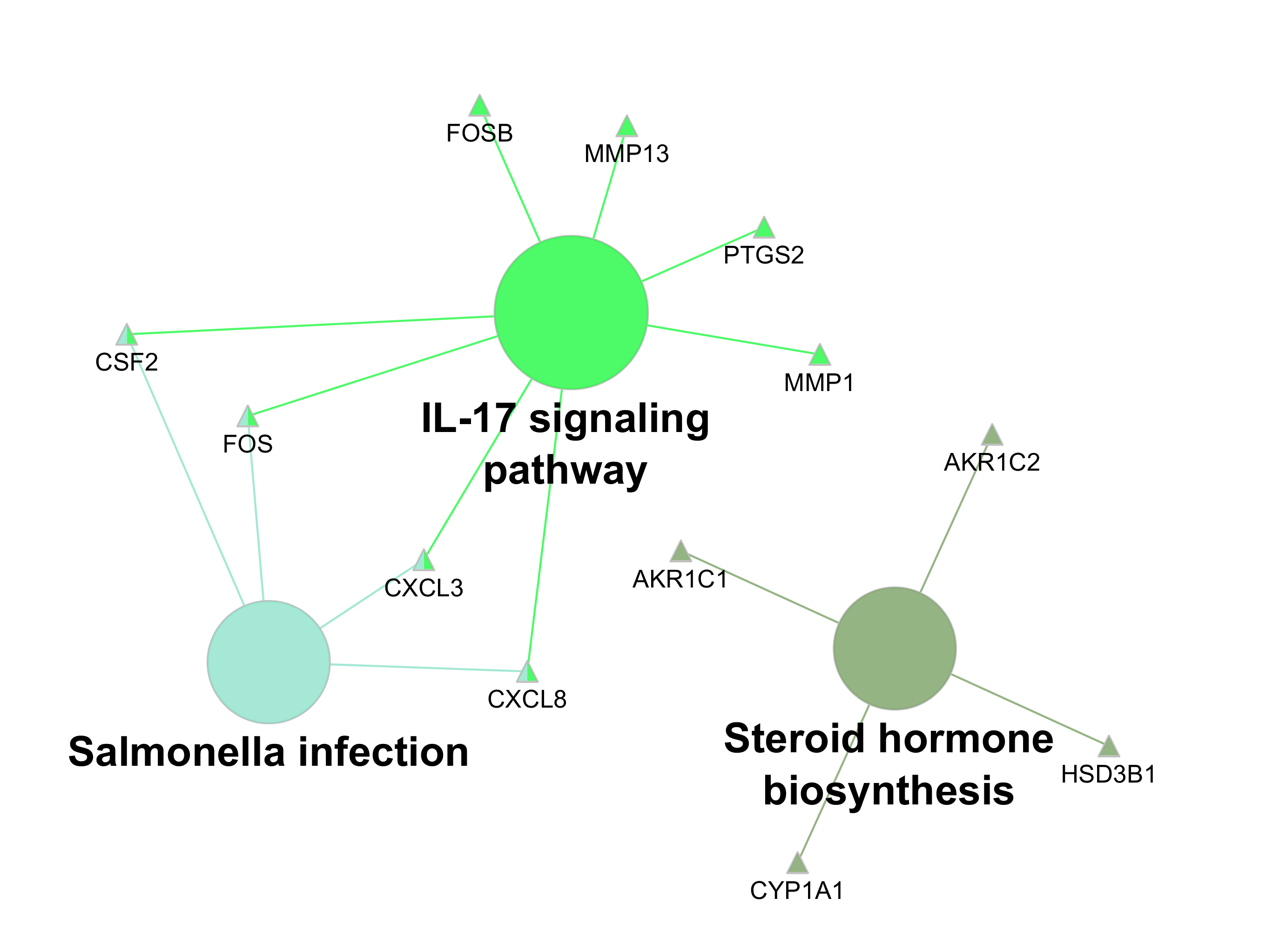

Supplement: Supplementary Figure 4 — Over-represented KEGG pathways and their contained genes showing shared DE genes between CKI (DE calculated by comparison to Kushen treated) and Kushen (DE calculated by comparison to untreated). Node size is proportional to the statistical significance of over-representation and genes are connected to their belonged pathways with edges. [file Image_4.PNG]
